# Modeling trajectories of physical aggression from infancy to pre-school age, their early predictors, and school-age outcomes

**DOI:** 10.1371/journal.pone.0291704

**Published:** 2024-06-03

**Authors:** Ane Nærde, Harald Janson, Mike Stoolmiller

**Affiliations:** 1 The Norwegian Center for Child Behavioral Development, Oslo, Norway; 2 Oregon Research Institute, Eugene, Oregon, United States of America; University of Lausanne: Universite de Lausanne, SWITZERLAND

## Abstract

This study identified latent trajectories of physical aggression (TPA) from infancy to preschool age and evaluated (a) effects of early parent, parenting and child predictors on TPA as well as on social, behavioral, and academic functioning in Grade 2, and (b) TPA effects net of early predictor effects on Grade 2 functioning. We used data from the Behavior Outlook Norwegian Developmental Study (BONDS), which included 1,159 children (559 girls). Parents reported on risk and protective factors, and on physical aggression from 1 to 5 years of age; teachers reported on Grade 2 outcomes. We employed latent class growth curve analyses and identified nine TPA. In fully adjusted models simultaneously testing all associations among predictors, trajectories, and outcomes, maternal and paternal harsh parenting, child gender, and sibling presence predicted TPA, which significantly predicted externalizing and academic competence in Grade 2. Child gender had a pervasive influence on all outcomes as well as on TPA. To our knowledge, this is the first trajectory study to determine which predictors are most proximal, more distal, or just confounded, with their relative direct effect sizes, and to link early paternal as well as maternal harsh parenting practices with children’s TPA. Our findings underscore the need to include fathers in developmental research and early prevention and intervention efforts.

## Introduction

In this study, we examined the developmental trajectories of physical aggression (TPA) during early childhood and explored how these patterns influence social, behavioral, and academic functioning in school aged children while simultaneously considering early individual and contextual risk and protective factors. While physical aggression is widely considered the most serious and socially unacceptable form of aggression, it is ontogenetically antecedent to less “serious” forms such as verbal or indirect aggression [1]. It has become increasingly evident that children’s use of physical aggression is normative during certain developmental periods. Behaviors such as kicking, hitting, and pushing originate in early childhood and have developmentally salient and adaptive functions.

Following previous research [2, 3], we use the term physical aggression to denote physical acts directed against others in the form of observable behaviors such as kicking and hitting, without consideration of intentionality. The systematic study of how physical aggression develops from early childhood onward is still recent [4], as most research has focused on aggression beginning in toddlerhood or at preschool age [but see e.g., 5, 6]. There is compelling evidence that (a) children start to physically aggress at an early age [7–9]; (b) such behavior typically peaks at approximately 2–3 years of age followed by a steep decline [5, 10–12]; and (c) a small proportion of children maintain a high and stable frequency of physical aggression during middle childhood that is associated with a range of negative outcomes [13–19].

### Developmental TPA across early childhood

We review research applying a developmental trajectory approach, that is, the identification of prototypic subgroup curves representing different developmental patterns of physical aggression (i.e., TPA) [e.g., 18]. We summarize key results from large-scale longitudinal studies exploring TPA starting in toddlerhood. In a seminal paper, Tremblay [1] reviewed the state of knowledge regarding the origins of aggressive behavior and highlighted the need to focus on the first few years of development. He argued that infants’ physical aggression typically has not been deemed developmentally significant and stated that “the exciting work that needs to be done is to study the developmental trajectories of these aggressive behaviors at the intra-individual level during infancy and toddlerhood and their relationships with later development” [1, p. 134]. Currently, the results from several longitudinal studies mapping the early development of physical aggression speak of the heterogeneity in this development [4, 15–16, 19, 20–22].

Research addressing TPA has found varying numbers of trajectories. This is likely related to sample variation, including variations in age range, sample size, risk factor composition, the number of repeated assessments, model specification, and selection criteria [10, 23]. Studies have typically identified three [10, 12, 19, 21], four [20, 24–28], or five trajectories [16, 22], and some report differing numbers of trajectories for girls and boys (i.e., 3 for girls and 5 for boys [4]). Consistent findings include at least one TPA showing stably low levels of aggression, and one TPA distinguished by a stably high level. Less consistent findings include a declining aggression trajectory [22, 25, 28]. No late-onset high physical aggression groups are usually identified. Despite strong calls [1, 23] to start early (by 6 to 8 months of age [7–8]), use multiple data sources, and include frequent observations (every 3 months [1]), existing evidence is typically based on a later start, maternal reports, and longer assessment intervals [4–5, 10, 19–20, 22, 28, but see e.g., 6, 8, 29].

### Predictors of TPA

Our focus is on environmental predictors of TPA and Labella and Mason [30] recently discussed the most salient family-based risk and protective factors for the development of aggression and violence, grouping them into *parent/family factors*, *parenting factors*, and *child factors*. In addition, it is important to acknowledge that twin and related research [31, 32] suggest substantial genetic or epigenetic factors [33, 34] related to TPA.

#### Parent/family factors

*Socioeconomic status (SES) and parental psychopathology*. Low SES and parental psychopathology are persistent predictors of child maladjustment, including physical aggression [30, 35–37]. In line with this, low household income [4, 10, 12, 20, 22], low parental education levels [4, 10, 20, 22], single parenthood [22], early maternal childbearing [3], and parental psychopathology [4, 12] are among the strongest predictors of belonging to the high-stable aggression group across trajectory studies. The family stress model [38] asserts that the stress of poverty and financial instability undermines parenting practices and affects subsequent child functioning.

*Siblings*. The presence of siblings (younger or older) constitutes a major risk factor for aggressive behavior [39–43]. Siblings represent easily accessible targets and intersibling aggression is the most prevalent form of family violence [44]. The relevant theoretical perspectives include family systems theory, evolutionary psychology, and social learning theories [43, 44]. For instance, Patterson [43, 45] suggested a sibling-training hypothesis wherein coercive interactions set the stage for the development and escalation of aggressive behavior through direct practice, modeling, and reinforcement.

#### Parenting factors

*Parenting style*. Less sensitive and involved maternal parenting has been linked with higher and more stable TPA from 2 years of age to the third grade [22], and maternal coercive parenting has been found to be predictive of TPA from 17 to 42 months of age [12], as well as from 2 to 8 years of age [20]. Patterson’s [45] coercion model asserts that aggression develops from negative reinforcement processes between a child and important others. This negative reinforcement for acting out is what perpetuates adverse parent–child cycles [45, 46].

The recurring failure to integrate fathers into developmental research, including research on aggression, may lead to the misattribution of paternal influences to maternal influences or leaving important sources of variation unexplained (for an exception, see Yang et al. [47]). Cabrera et al. [48] and Cabrera [49] recently discussed why fathers are still mostly absent from parenting research despite strong suggestions that traditional models of developmental influences are outdated; one reason includes the tendency to define mothers as the primary caregiver and the belief that fathers do not engage in hands-on parenting. Norwegian fathers tend to be highly involved in caregiving [50], providing a unique opportunity to assess the importance of factors related to mothers and fathers alike (e.g., parenting style and psychopathology) in the development of aggression.

#### Child factors

*Gender*. Whereas empirical evidence suggests that boys and girls use physical force against others (e.g., pulling hair, hitting) at similar rates in infancy and early toddlerhood [7, 51, 52], boys show significantly more physical aggression in the following few years [5, 7, 26, 53]. Côté et al. [20] reported that children in the high TPA from 2 to 8 years were more likely to be boys. Multiple hypotheses for these differences have been proposed [20, 24, 51–54], including a convincing case for a developmental cascade of biological and social factors [51].

*Temperament*. High negative emotionality, activity levels and reactivity, along with low effortful control and negative mood, have all been linked to TPA [26, 29, 55, 56]. Several theoretical models go beyond simple associations to postulate synergistic interactions between difficult temperaments (i.e., high negative emotionality, activity level, and reactivity) and negative parenting that influence TPA [57–59], and a recent meta-analysis provides partial support [60] for these theories.

### Outcomes of TPA

The National Institute of Child Health and Human Development Study of Early Child Care and Youth Development [SECCYD; 15, 22] showed that both level and stability of aggression from age 2 to 9 years of age predict middle childhood academic achievement and social functioning at home, at school, and in the peer groups. In particular, Campbell et al. [15] explored adjustment from 9 to 12 years of age as a function of five identified trajectories and found that children in the high- and moderate-stable groups showed continuing behavioral and social problems relative to those in the very low aggression group; the high-stable group showed signs of significant antisocial behavior, whereas the moderate-stable group showed symptoms of ADHD and more minor peer problems [15, 22]. Additionally, children in the moderate decreasing group displaying transient and age-related aggression that disappeared before school age showed good adjustment both at the age of 9 years [22] and across the transition to adolescence [15]. A more surprising finding was that relative to those in the very-low group, children in the low-stable group had elevated scores on measures of problem behaviors and poor social skills in elementary school [15]. Thus, moderate, or low-level aggression that persists from toddlerhood through school entry may signal adjustment problems that will continue or emerge by middle childhood.

### The present study

To the best of our knowledge, this is the first study of TPA with the following combination of characteristics: a large population-based sample, frequent repeated measures, and an age range of 1–5 years, during which physical aggression both emerges and declines among most children. This study also extends previous findings by (a) including father parenting and other paternal characteristics, (b) using a North European sample, and (c) utilizing recent advances in statistical methods when incorporating predictors and outcomes in the trajectory model [61]. We included 20 early predictors and four school-age outcomes suggested by prior research, enabling us to disentangle the relative importance of TPA for social, behavioral, and academic functioning, while simultaneously considering the direct and indirect impacts of early risk and protective factors.

### Research objectives and expectations

Our specific research objectives were to:

1. Model trajectories of physical aggression (TPA) from 1 to 5 years of age;
2. Predict TPA membership from a set of 20 early predictors and predict Grade 2 outcomes (externalizing, internalizing, social skills, and academic competence) from TPA;
3. Test all simultaneous influences in fully adjusted models, allowing us to determine predictors of TPA and Grade 2 outcomes that remained significant after adjustment for other competing predictors.

The expectations we had concerning findings with regard to our three respective objectives were as follows:

1. Our findings would generally agree with those from previous research concerning TPA shapes and distributions, however; due to the dataset’s frequent repeated measures and the eventful age range, our research might identify more distinct TPA compared to prior research;
2. Previously identified early risk and protective factors, considered individually, would predict TPA and Grade 2 outcomes consistent with prior research; and
3. Fewer early predictors of TPA and Grade 2 outcomes would remain significant in the fully adjusted models; however, TPA would remain a significant predictor of Grade 2 outcomes. We did not have clear expectations concerning which predictors would retain direct effects or work indirectly in predicting Grade 2 outcomes.

## Materials and methods

### Sample and procedures

We utilized data from the Behavior Outlook Norwegian Developmental Study (BONDS), a population-based longitudinal study of 1,159 children (559 girls and 600 boys) from five municipalities in southeast Norway conducted by the Norwegian Center for Child Behavioral Development. The BONDS was approved by the Regional Committee for Medical and Health Research Ethics and the Norwegian Social Sciences Data Services (approval numbers S-06067; 2009/224a), and all parents provided informed written consent. Participants were recruited from 2006 to 2008 during the 5-month visit at child health clinics, which are attended almost universally. The inclusion criteria were the child being of the appropriate age and one parent being able to participate without an interpreter.

Statistical power considerations, including of growth mixture models, guided the determination of the sample size [62]. Families of 1,931 eligible children were informed about the project. Of the 1,465 (76%) families who agreed to be contacted, 1,159 (79%, or 60% of the eligible group) opted to participate [62]. Details of the BONDS sample and procedures including a flow chart of participation have been previously published [62, 63].

The original study comprised parental interviews, including a computerized questionnaire, when the child was aged 6 months and 1, 2, 3, and 4 years. Whereas both parents were invited to participate in the first interview, fathers were primarily targeted in the 1- and 3-year waves, and mothers were targeted in the 2- and 4-year waves. At the ages of 1, 2, and 3 years, the assessment included videotaped structured parent–child interaction tasks, in which more fathers participated when the child was aged 1 and 3 years, and more mothers participated when the child was aged 2 years. In addition to the yearly data collection, brief telephone interviews comprising a selection of items relevant to the study’s key outcomes were conducted when the child was aged 8 months, 10 months, and 1.25, 1.50, 1.75, 2.33, 2.67, and 3.5 years, as well as before and after entry into center-based childcare.

At the age of four years, the parents of 1,027 children (88.6%) renewed their informed written consent for participation in a continuation of the study, including parental interviews when the child was aged 5 years and in Grades 1 and 2 and teacher-completed questionnaires when the child was in Grades 1 and 2.

### Measures

#### Child physical aggression

We used an original instrument developed for the BONDS, with idiomatic wording and a selection of items widely acceptable to parents of small children, including exclusively physical behavior directed at others covering mostly the lower range of intensity. The items covered hitting, pushing, pulling hair, pinching, throwing things, biting, and kicking. In questionnaires completed by parents when the child was aged 1, 2, 3, and 4 years, a seven-point frequency response scale ranging from 1 (*never/not in the past year*) to 7 (*three times daily or more*) was used. In telephone interviews conducted in-between the questionnaires and at 5 years of age, the response format was dichotomous (*yes/no*). We used all available reports from either questionnaires or telephone interviews from 1 to 5 years of age, with an average of twelve repeated measures per child (ranging from 1 to 21, with 90% of children having at least ten reports). The full instrument is reproduced in S1 Appendix, along with item-level descriptive statistics.

We employed Rasch scaling [64] to equate the questionnaire and telephone-interview response formats, link response patterns with varying sets of completed items, and address measurement invariance. We used Rasch person location estimates as our physical aggression measure (*M* = -1.86; SD = 1.87). Specifics of the scaling and further statistics of the Rasch-scaled measure are given in S2 Appendix. A simple, nonparametric regression (Loess) plot of the repeated measures of physical aggression vs. child age is shown in S1 Fig.

#### Parent/family predictor measures

All early predictor variables were obtained at either the 6- or 12-month assessments. In addition to interview self-reports of **single-parent status**, for **young mother/young father** we used a binary indicator of young parent age (i.e., ≤25 years old).

We used interview reports of **parental education** in six categories of total years of education.

**Financial stress** was measured via a binary (yes/no) parental questionnaire self-report of having long-standing financial difficulties in the past year (e.g., rent, mortgage).

For **couple relationship** we used mothers’ questionnaire reports of the quality of the relationship with her partner on a shortened and modified 10-item version of the Relationship satisfaction scale [65] answered on a 6-point Likert scale from 1 (*very much agree*) to 6 (*very much disagree*) with Cronbach’s alpha of .90 in our sample.

Information on **parental depression and anxiety** was obtained using the 12-item version of the Hopkins Symptom Check List [66] self-report questionnaire, a subset of the 25-item version [67]. Items are scored using a Likert-type scale ranging from 1 (*not at all*) to 4 (*extremely*). Sample Cronbach’s alphas for the mean scores were .88 and .87 for mothers and fathers, respectively.

#### Parenting predictor measures

*Parent sensitivity*. Parent sensitivity was measured based on observer ratings of videotaped parent–child structured interaction tasks when the child was aged 1 year. We used the mean of three ratings–sensitivity/responsiveness, detachment/disengagement (reversed), and positive regard for the child–from the Norwegian adaptation of the Qualitative Ratings for Parent-Child Interactions used in the SECCYD study [68, 69]. Ratings were aggregated across two separately rated segments of interaction. Each variable was rated by trained judges on a Likert-type scale ranging from 1 to 5; interrater reliability (intraclass correlations) ranged from .65 to .74 in the full set of rating variables [69]. Internal consistency (alpha) for the composite was .88 (fathers) and .86 (mothers). The parent sensitivity measure is detailed in S3 Appendix.

*Harsh and positive parenting*. When the child was aged 1 year, the parents contributed questionnaire self-reports of parenting practices on a subset of items from the Parenting scale from the Fast Track Project; this scale was adapted from the Parental Discipline scale and Parent Praise scales from the Pittsburgh Youth Study [70, 71]. The items were answered on a frequency scale ranging from 1 (*never*) to 5 (*almost always*). We used the mean of three items on **positive** parenting behavior (smiling at the child, saying something nice about the child to the child, and giving the child a hug, pat on the back, or a kiss) in response to desirable child behavior. We used the mean of two items on **harsh** parenting behavior (yelling or screaming and slapping or hitting) in response to undesirable child behavior. Reliabilities were estimated at .60, .58, .86, and .81 for fathers’ harsh, mothers’ harsh, fathers’ positive, and mothers’ positive parenting, respectively.

#### Child predictor measures

In addition to child **gender** and **age**, the presence of **similar-aged sibling**(s) was a binary indicator of interview reports of the presence of a sibling with an age difference of up to 5 years.

For child temperament we used parent questionnaire reports completed when the child was aged 6 months including the **Activity level** and **Distress to Limitations** scales from Rothbart’s Infant Behavior Questionnaire-Revised [72], as well as the **Soothability** scale from the Infant Behavior Questionnaire [73]. A modified three-point response format was used: 1 (*most of the time*), 2 (*sometimes*), or 3 (*rarely or never*). Cronbach’s alphas were .71, .76, and .77 for the three scale mean scores, respectively.

#### Grade 2 outcome variables

Teachers completed the Social Skills Improvement System-Rating Scales (SSIS-RS; [74]). The children were approximately 8 years old around the time of data collection. **Externalizing, internalizing, academic competence**, and **social skills** were evaluated using sums of 12, 7, 7, and 46 items, respectively, with sample Cronbach’s alphas of .92, .83, .96, and .96, respectively. The scales use a four-point response format ranging from 0 (*never*) to 3 (*almost always*), except academic competence, which is rated from 1 (*lowest 10%*) to 5 (*highest 10%*).

### Statistical analyses

#### Analytic subsample and missing data

The present study used an analytic subsample of 1141 children with reports of physical aggression. Among predictor variables from 6 months of age representing family demographics and child temperament, 1.3% missing observations were imputed prior to the main analyses using a single imputation of missing values, as described in more detail in Nærde et al. [11]. For the remaining early predictor variables and Grade 2 outcomes, missingness was addressed using 420 multiple imputations in R [100] for standard regression analyses. The imputation model included all early predictors and Grade 2 outcomes. For all analyses involving latent classes, missingness was handled within the latent-variable framework in Mplus as described below in the Analytic strategy subsection.

Attrition analyses revealed that the analytic subsample and the subsample of 901 children who had Grade 2 teacher reports resembled the original study sample very closely with respect to all early predictors (S4 Appendix).

#### Data transformations

To prevent potential distortions due to outliers, we trimmed nonbinary predictors at approximately the 2nd and 98th percentiles of their respective distributions. Some of the nonbinary predictors as well as the Grade 2 outcomes of externalizing and internalizing were highly skewed, as would be expected in this population, and were square root transformed to make the assumption of within-class normality more reasonable. Descriptive statistics for predictors and outcomes, before and after transformations, are shown in Table 1.

**Table 1:** Descriptive statistics.

		Raw data						Transformed data					
	*N*	*M*	*SD*	Skew	Kurt	Min	Max	*M*	*SD*	Skew	Kurt	Min	Max
		Trajectory (growth model) repeated measure											
**Child physical aggression**	13673	-1.86	1.87	0.28	-0.23	-5.30	5.02	—	—	—	—	—	—
		Early predictors											
**Maternal education** ^a^	1141	14.29	2.57	-0.14	-1.06	9	18	0.00	2.57	-0.14	-1.06	-5.29	3.71
**Paternal education** ^a^	1141	13.84	2.59	0.23	-1.09	9	18	0.01	2.59	0.23	-1.09	-4.84	4.17
**Young mother**	1141	0.14	0.34	2.12	2.49	0	1	—	—	—	—	—	—
**Young father**	1141	0.06	0.23	3.86	12.95	0	1	—	—	—	—	—	—
**Financial stress**	1096	0.12	0.33	2.34	3.46	0	1	—	—	—	—	—	—
**Couple relationship** ^b^	1072	5.33	0.65	-1.70	4.18	1.20	6.00	0.70	0.39	-0.15	-0.70	0.00	1.38
**Single parent**	1141	0.05	0.21	4.32	16.66	0	1	—	—	—	—	—	—
**Mat’l depression & anxiety** ^c^	1116	1.33	0.37	1.95	5.98	1.00	3.75	0.47	0.32	0.07	-0.82	0.00	1.15
**Pat’l depression & anxiety** ^c^	671	1.27	0.33	2.59	13.12	1.00	4.00	0.40	0.31	0.10	-1.11	0.00	1.00
**Maternal sensitivity** ^d^	244	3.90	0.61	-0.79	0.36	1.83	5.00	0.01	0.59	-0.63	-0.31	-1.43	0.93
**Paternal sensitivity** ^d^	717	3.74	0.69	-0.45	-0.17	1.17	5.00	0.00	0.66	-0.32	-0.75	-1.41	1.09
**Mat’l harsh parenting** ^d^	377	1.42	0.50	1.96	7.73	1.00	5.00	-0.01	0.47	1.28	1.70	-0.42	1.58
**Pat’l parenting** ^d^	835	1.40	0.47	1.23	1.77	1.00	4.00	-0.01	0.44	0.82	-0.28	-0.41	1.10
**Mat’l positive parenting** ^d^	377	4.71	0.45	-2.82	13.90	1.00	5.00	0.01	0.40	-1.61	2.25	-1.38	0.29
**Pat’l positive parenting** ^d^	835	4.53	0.54	-2.10	8.50	1.00	5.00	0.02	0.47	-0.86	-0.18	-1.19	0.47
**Child gender**	1141	0.52	0.50	-0.06	-2.00	0	1	—	—	—	—	—	—
**Similar-aged sibling**	1141	0.38	0.49	0.47	-1.78	0	1	—	—	—	—	—	—
**Child activity level** ^d^	1141	2.07	0.28	0.07	0.00	1.09	2.83	0.00	0.27	0.11	-0.42	-0.54	0.60
**Child distress to limitations** ^d^	1141	1.69	0.31	0.40	0.13	1.00	2.82	0.00	0.30	0.30	-0.31	-0.56	0.70
**Child soothability** ^d^	1141	2.63	0.29	-0.46	-0.35	1.56	3.00	0.00	0.28	-0.30	-0.89	-0.63	0.37
		Grade 2 outcomes											
**Externalizing** ^e^	901	4.81	5.26	1.68	3.22	0	35	1.83	1.21	0.34	-0.36	0.00	5.92
**Internalizing** ^e^	901	3.01	2.97	1.17	1.49	0	18	1.42	1.00	-0.04	-0.94	0.00	4.24
**Academic competence**	896	25.03	7.12	-0.40	-0.50	7	35	—	—	—	—	—	—
**Social skills**	887	103.79	19.56	-0.43	-0.45	32	137	—	—	—	—	—	—

Note. Some variables were transformed as marked by letter subscripts. Descriptive statistics for transformed variables are displayed in the right part of the table. Reversing and recentering are linear transformations that do not change the shape of a distribution, the rank order of observations, or the linear relations with other variables. Trimming and square root transformations are non-linear transformations that change the shape of a distribution (make it less skewed and heavy-tailed), the rank order of a few of the observations (only trimming and this is because it produces ties among extremes) and thus potentially alter linear relations with other variables.

^a^Centered.

^b^Trimmed (values smaller than the 2nd percentile or larger than the 98th percentile recoded back to the 2nd or 98th percentile values), reversed, recentered to have a minimum score of zero and square root transformed.

^c^Trimmed, recentered to have a minimum score of zero and square root transformed.

^d^Centered and trimmed.

^e^Square root transformed.

### Analytic strategy

*Number and shapes of TPA*. First, we used latent class trajectory analysis (LCGA) to investigate the number and shape of TPA. We specified TPA as a 4th-degree (quartic) polynomial growth model, following preliminary analyses that indicated that this model fit substantially better than 2nd- or 3rd-degree models in our sample. We used a 2-level specification for the LCGA with the child’s age in years, centered at 2.2, as the time variable to accommodate the large number and irregular timing of the repeated growth measures within children. The basic 2-level LCGA-growth model specification included 5 child-level growth components with freely estimated means for the 5 growth components in each class, no within-class variability in growth components, and time-specific variability constrained to be equal across both time and classes. The specification of no within-class variability in growth is what locates our model in the category of LCGA (latent class growth analysis; [18]), which is considered the simplest longitudinal mixture model [75]). Most prominent trajectory studies to date, [e.g., 10, 18, 22, 26] have employed LCGA models.

*Unadjusted predictor effects on TPA membership*. After selecting the optimal TPA solution, in the following steps, for models that included latent classes and either predictors, outcomes, or both, we used the manual 3-step procedure as recommended by Asparouhov and Muthen [61]. In addition to the advantages given by Asparouhov and Muthen, the 3-step approach allows a much simpler way of dealing with missing data in child-level predictors because Step 3 eliminates all the repeated growth measurements for a particular child and replaces them with one child-level nominal predictor of latent class, that is, pseudo-class.

We first examined all prediction effects unadjusted for other influences. In the first of these steps, we considered early predictors of class membership. For the overall effect of a predictor on class membership, we used likelihood ratio tests (LRT) to compare the model with all possible predictor effects to the model that included the predictor but had all predictor effects constrained to zero. We used a pseudo-*R* statistic [76] as the indicator of the strength of association between predictor and class membership. We also investigated predictor effects on membership in any class versus another in multinomial logistic regressions in the context of the 3-step approach. Multinomial logistic contrast coefficients express the change in log odds of the outcome for a 1-unit change in the predictor. In our case with 9 latent classes, the multinomial logistic regression produces 8 effects for each class vs. the reference class for each predictor. The 8 effects can be recombined in simple linear combinations to derive any contrast between any 2 classes.

*Unadjusted effects of TPA membership on Grade 2 outcomes*. Next, we evaluated the overall effect of class membership on the distal outcomes considered singly. Similar to our approach for comparing predictor effects, we adopted an LRT approach comparing the model with distal outcome means freely estimated in each class to the null model with distal outcome means forced to be equal in each class. To summarize the overall effect of latent class on outcomes, we computed *R* as the square root of *R*^*2*^ by computing the proportional reduction of total outcome variance represented by the residual, within class outcome variance. For means comparisons of outcomes among latent classes, we used standardized mean differences.

*Unadjusted predictor effects on Grade 2 outcomes*. Next, we examined the strength of association between each early predictor and each Grade 2 outcome using a standardized regression weight.

*Fully adjusted models predicting each distal outcome from early predictors and TPA*. Finally, for each distal outcome, we went on to build fully adjusted models, that simultaneously considered the influence of early predictors on TPA as well as the influence of TPA and early predictors on the outcome. For these analyses, we retained those early predictors that either had a significant unadjusted effect on TPA membership or had a significant effect on any of the four distal outcomes after adjusting for all other predictors in separate multi-predictor models. The fully adjusted models included all possible predictor effects on latent class, all latent class effects on the distal outcome, and all possible direct paths from the predictors to the distal outcome. Analogous to the unadjusted-model analyses, we investigated effects using the LRT, pseudo-*R* statistics, and multinomial logistic contrast coefficients.

## Results

### Number and shapes of TPA

**Fig 1:**
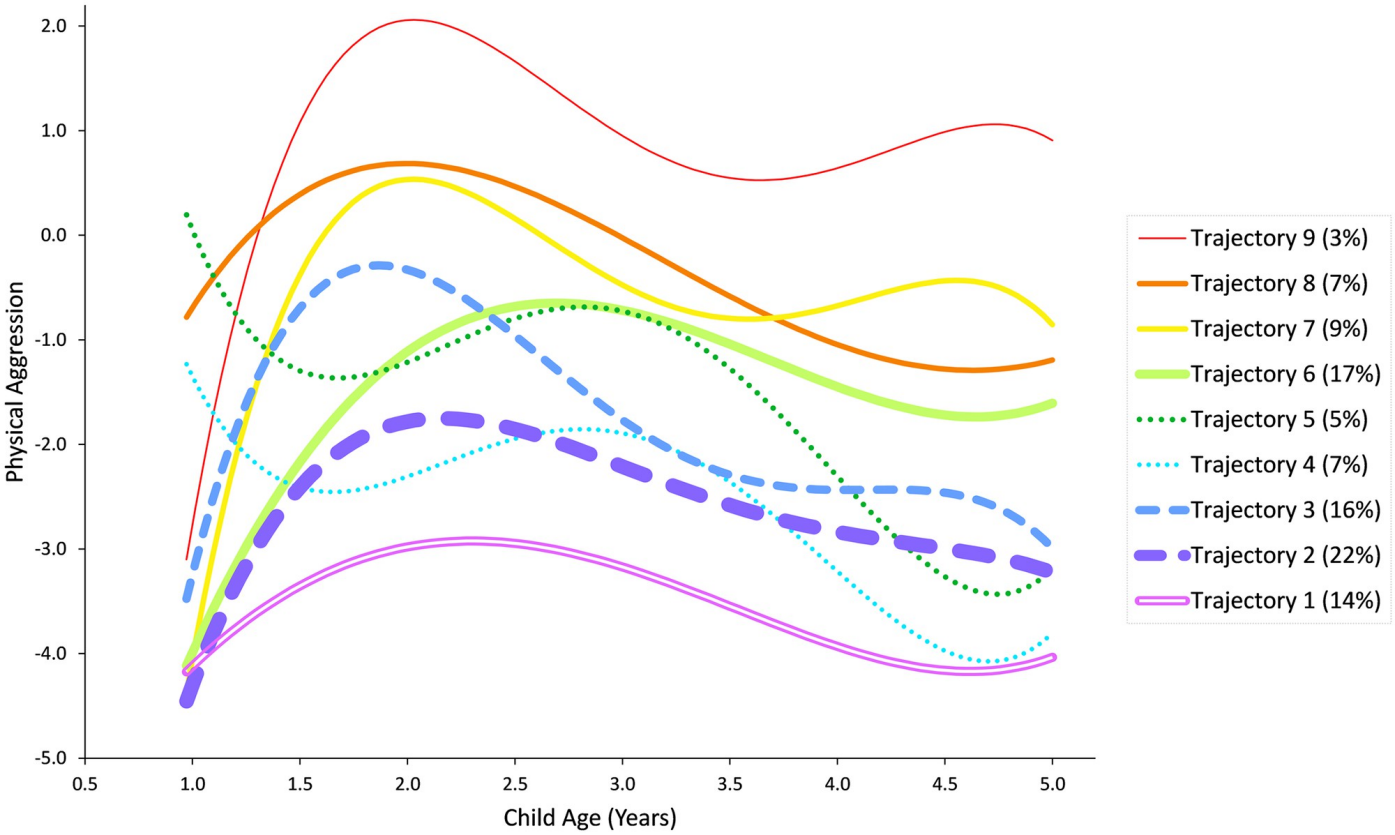
Model predicted child physical aggression by trajectory in nine-trajectory solution (fourth-grade polynomial growth function).

We considered two- to ten trajectory solutions. We relied on information criteria, the parametric bootstrap likelihood ratio test (BLRT), the test that has the best support mathematically [77, 78] and in simulations [79], and substantive considerations to select the number of TPA that seemed optimal with respect to parsimony, model fit, the distinctiveness of the trajectories, and the estimated proportions of the population within each trajectory. S5 Appendix provides information criteria and BLRT for the sequence of the models. The consistent Akaike information criterion (CAIC; [79]) was minimized by eight trajectories, the Bayesian information criterion (BIC) was minimized by nine trajectories, and the sample size-adjusted BIC and the Akaike information criterion (AIC) did not reach a clear minimum by ten trajectories. The BLRT consistently rejected models with fewer than 8 trajectories, although due to the heavy computational burden we did not attempt to test models with 9 or more trajectories against simpler models. We selected the nine-trajectory solution over the eight because it was suggested by the BIC, which is strongly recommended by methodologists as being accurate, or at worst somewhat conservative, in this context [79], and suggested possibly distinct developmental paths worthy of investigation, each representing a reasonable proportion of the sample. Fig 1 shows the 9-class model estimated trajectories, which seemed to represent:

1. One *high-stable* trajectory that rises to a very high peak of physical aggression at approximately 2 years of age and stays at a high level (Trajectory 9, model-based trajectory proportion 3%);
2. Three *intermediate-endpoint* trajectories that end up at an elevated level of physical aggression, with different early developments: one starting high and declining after a rather high peak (Trajectory 8, 7%), another with a similar peak and decline but a low starting point (Trajectory 7, 9%), and a third starting low with a late and flat peak (Trajectory 6, 17%);
3. Two *high-start*, *low-endpoint* trajectories that decline from an initial high level, with a late and flat peak followed by another decline to a low level, with one trajectory following a slightly higher-level path (Trajectory 5, 5%) than the other (Trajectory 4, 7%);
4. Two *medium-peak*, *low-endpoint* trajectories that start and end with a low level of physical aggression, with one trajectory reaching an early peak of a somewhat high level (Trajectory 3, 16%), and one with a flatter peak at a lower level (Trajectory 2, 22%); and
5. One *low-stable* or "*no aggression*" trajectory that starts and stays at a low level of aggression with just a minor rise to a very low, flat peak, and a decline back to a low level (Trajectory 1, 14%).

Details of the 9-trajectory solution are given in S6 Appendix.

### Unadjusted predictor effects on TPA

Overall effects of each predictor in isolation on TPA are shown in the first three data columns of Table 2.

**Table 2:** Unadjusted overall prediction effects of early predictors on trajectory and outcomes, and of trajectory on outcomes.

Predictor	Prediction to trajectory^a^			Prediction to Grade 2 outcomes^b^			
	χ^2^	*p*	Pseudo *R*^a^	Externalizing	Internalizing	Academic competence	Social skills
**Maternal education**	17.1	.029	.12*	-.13***	-.12***	.22***	.18***
**Paternal education**	14.5	.070	.11	-.12***	-.09**	.17***	.15***
**Young mother**	13.7	.090	.11	.09**	.11**	-.01	-.09*
**Young father**	5.9	.660	.07	.09*	.13***	-.05	-.08*
**Financial stress**	12.2	.144	.10	.16***	.15***	-.13***	-.18***
**Couple relationship (reversed)**	23.3	.003	.14**	.09*	.12***	-.08*	-.06
**Single parent**	13.9	.086	.11	.16***	.13***	-.14***	-.15***
**Maternal depression & anxiety**	40.8	< .001	.19***	.07*	.09**	-.09**	-.06
**Paternal depression & anxiety**	16.1	.041	.12*	.13**	.08	.00	-.17***
**Maternal sensitivity**	4.4	.818	.06	-.12	-.13*	.11	.09
**Paternal sensitivity**	11.4	.179	.10	-.12**	-.07	.10*	.11**
**Maternal harsh parenting**	46.5	< .001	.20***	.13*	.04	-.09	-.09
**Paternal harsh parenting**	31.7	< .001	.17***	.06	.01	-.11**	-.09*
**Maternal positive parenting**	6.5	.594	.08	.02	-.02	-.06	-.03
**Paternal positive parenting**	7.5	.485	.08	.03	.04	.04	-.02
**Child gender**	54.7	< .001	.22***	.30***	.13***	-.13***	-.30***
**Similar-aged sibling**	66.6	< .001	.24***	-.02	-.07*	-.02	.04
**Child activity level**	32.3	< .001	.17***	.09**	.09**	-.03	-.08*
**Child distress to limitations**	38.0	< .001	.18***	-.04	.00	.02	.04
**Child soothability**	29.5	< .001	.16***	.09**	.03	-.07*	-.11**
**Trajectory** ^c^	—	—	—	.27***	.11	.19***	.19**

*p < .05

**p < .01

***p < .001.

^a^Overall prediction to trajectory was assessed by comparing a model with all class discrimination effects of each single predictor freely estimated to a reduced model where all the class discrimination effects are zeroed out in an 8 degrees of freedom chi-square test; the effect size is given as pseudo-*R* derived from robust chi-square estimated as the square root of Nagelkerke’s pseudo *R*^*2*^, which was identical to Cohen’s ω.

^b^Predictor-to-outcome associations are given as fully standardized regression coefficients. Coefficients were standardized by sample standard deviations (Table 1).

^c^Prediction of outcomes from trajectory membership is given as *R*, the square root of *R*^*2*^ computed as the proportional reduction in total outcome variance represented by the residual within class variance, that is, *R*^*2*^ = proportion explained variance = 1 - (residual variance/total variance).

Eleven out of the 20 predictors had significant overall effects on TPA with pseudo-*R* values ranging from .12 to .24. The presence of a similar-aged sibling and child gender were the two strongest predictors. Maternal education level, couple relationship, depression and anxiety in both parents, harsh parenting by both parents, and all three child-temperament variables were also significant predictors. Trajectory predictor coefficients with pairwise contrast tests are shown in the upper part of Table 3. In the presence of a significant overall effect, pairwise contrast tests may aid the interpretation of effect direction. In the absence of a significant overall effect, the presence of any effects notable in size but limited in scope—that is, affecting only a few classes or small classes—cannot be inferred from the displayed contrast tests alone but must be considered in the context of the magnitude of the contrast.

**Table 3:** Trajectory predictor coefficient contrasts for unadjusted effects of early predictors on trajectory and means comparison contrasts for unadjusted effects of trajectory on outcomes.

Variable	Overall effect^a^	Trajectory								
		1	2	3	4	5	6	7	8	9
Trajectory prediction coefficients with pairwise contrasts for effects of early predictors on trajectory^b^										
**Maternal education**	.12*	0.00	0.15^4^,^8^,^9^	0.00	-0.27^2^,^6^,^7^	0.12	0.13^4^,^8^,^9^	0.21^4^,^8^,^9^	-0.24^2^,^6^,^7^	-0.40^2^,^6^,^7^
**Paternal education**	.11	0.00^9^	-0.04	-0.10	-0.17	-0.02	-0.03^9^	0.21^8^,^9^	-0.31^7^	-0.60^1^,^6^,^7^
**Young mother**	.11	0.00	-0.02	0.03	0.23^6^,^7^	-0.04	-0.29^4^,^8^	-0.30^4^	0.12^6^	-0.03
**Young father**	.07	0.00	-0.03	-0.07	-0.08	-0.07	-0.23^9^	-0.17	-0.06	0.18^6^
**Financial stress**	.10	0.00^9^	0.04^9^	0.20	0.29	0.23	0.09^9^	0.26	0.29	0.46^1^,^2^,^6^
**Couple relationship (reversed)**	.14**	0.00^6^	-0.13^3^,^5^,^6^,^8^,^9^	0.21^2^,^4^	-0.22^3^,^6^,^8^,^9^	0.23^2^	0.28^1^,^2^,^4^	0.17	0.24^2^,^4^	0.45^2^,^4^
**Single parent**	.11	0.00	-0.50	0.02	0.06	-0.12	-0.05	0.12	0.15	0.06
**Maternal depression & anxiety**	.19***	0.00^3^,^6^,^7^,^8^,^9^	0.09^3^,^7^,^8^,^9^	0.45^1^,^2^	0.30^9^	0.33	0.32^1^,^9^	0.57^1^,^2^	0.63^1^,^2^	0.84^1^,^2^,^4^,^6^
**Paternal depression & anxiety**	.12*	0.00^8^	-0.35^4^,^8^	0.05	0.29^2^	0.04	0.15	0.10	0.47^1^,^2^	0.18
**Maternal sensitivity**	.06	0.00	0.09	-0.11^5^	-0.09^5^	0.68^3^,^4^,^6^,^7^,^8^	-0.07^5^	-0.10^5^	-0.08^5^	0.22
**Paternal sensitivity**	.10	0.00	-0.03	-0.13^4^	0.57^3^,^5^,^6^,^9^	-0.22^4^	-0.24^4^	0.08	0.03	-0.22^4^
**Maternal harsh parenting**	.20***	0.00^3^,^8^	-0.64^3^,^4^,^5^,^6^,^7^,^8^,^9^	0.88^1^,^2^	0.68^2^	0.72^2^	0.65^2^,^8^	0.64^2^	1.15^1^,^2^,^6^,^9^	0.47^2^,^8^
**Paternal harsh parenting**	.17***	0.00^3^,^6^,^7^,^9^	0.23^7^,^9^	0.51^1^,^9^	0.45^9^	0.48^9^	0.54^1^,^9^	0.75^1^,^2^	0.47^9^	1.00^1^,^2^,^3^,^4^,^5^,^6^,^8^
**Maternal positive parenting**	.08	0.00	-0.19	-0.43	-0.10	-0.46	-0.13	-0.38	-0.39	0.00
**Paternal positive parenting**	.08	0.00^2^,^8^	-0.43^1^	-0.29	-0.35	-0.25	-0.22	-0.21	-0.43^1^	-0.46
**Child gender**	.22***	0.00^2^,^3^,^4^,^5^,^6^,^7^,^8^,^9^	0.59^1^	0.59^1^	0.47^1^,^7^	0.73^1^	0.80^1^	0.90^1^,^4^	0.76^1^	0.98^1^
**Similar-aged sibling**	.24***	0.00^3^,^5^,^6^,^7^,^8^,^9^	0.33^7^,^8^,^9^	0.37^1^,^7^,^8^,^9^	0.07^6^,^7^,^8^,^9^	0.55^1^	0.53^1^,^4^,^7^,^9^	0.90^1^,^2^,^3^,^4^,^6^	0.80^1^,^2^,^3^,^4^	1.00^1^,^2^,^3^,^4^,^6^
**Child activity level**	.17***	0.00^4^,^5^,^8^,^9^	-0.04^4^,^5^,^8^,^9^	0.06^4^,^8^	0.45^1^,^2^,^3^,^6^	0.35^1^,^2^,^6^	-0.09^4^,^5^,^8^,^9^	0.18	0.50^1^,^2^,^3^,^6^	0.37^1^,^2^,^6^
**Child distress to limitations**	.18***	0.00^3^,^4^,^5^,^6^,^7^,^8^,^9^	0.32^5^,^8^	0.56^1^	0.63^1^	0.75^1^,^2^	0.53^1^	0.54^1^	0.68^1^,^2^	0.67^1^
**Child soothability**	.16***	0.00^2^,^3^,^5^,^6^,^8^	-0.34^1^,^8^	-0.39^1^,^8^	-0.17^8^	-0.58^1^	-0.45^1^,^8^	-0.20^8^	-0.76^1^,^2^,^3^,^4^,^6^,^7^,^9^	-0.29^8^
Standardized means comparisons with pairwise contrasts for effects of trajectory on distal outcomes^c^										
**Externalizing**	.27***	0.00^2^,^3^,^4^,^5^,^6^,^7^,^8^,^9^	0.32^1^,^6^,^7^	0.37^1^,^7^	0.47^1^,^7^	0.48^1^,^7^	0.71^1^,^2^	0.99^1^,^2^,^3^,^4^,^5^	0.63^1^	0.75^1^
**Internalizing**	.11	0.00	0.06	0.19	0.25	-0.03	0.16	0.17	0.31	0.46
**Academic competence**	.19***	0.00^9^	-0.19^9^	-0.24^9^	-0.11^9^	0.08^7^,^9^	-0.19^9^	-0.34^5^,^9^	-0.38^9^	-1.09^1^,^2^,^3^,^4^,^5^,^6^,^7^,^8^
**Social skills**	.19**	0.00^6^,^7^,^9^	-0.06^6^,^7^,^9^	-0.15^9^	-0.43	-0.35	-0.42^1^,^2^	-0.45^1^,^2^	-0.33	-0.80^1^,^2^,^3^

*p < .05

**p < .01

***p < .001 for overall effects.

^a^Pseudo-*R*/*R* estimate with overall significance test from Table 2.

^b^Trajectory prediction contrast coefficients are fully standardized logistic regression effects on the log odds scale (the effect on the log odds of the class contrast for a change in the predictor of one standard deviation) given relative to membership in Trajectory 1 (the "no aggression" trajectory). Coefficients were standardized by sample standard deviations (Table 1). Contrast coefficients between any pair of trajectories can be derived by subtraction (i.e., for maternal education between Trajectories 2 and 4: 0.15- [-0.27] = 0.42, which is significant at *p* < .05 as indicated by superscripts). A contrast coefficient of 0.69 is equivalent to an odds ratio of 2.0, and a coefficient of -0.69 is equivalent to an odds ratio of 0.5.

^c^The entries are standardized mean differences relative to Trajectory 1.

^123456789^Number superscripts indicate pairwise contrast tests significant at *p* < .05 between the indexed trajectory and the trajectory with the superscripts’ number; significant contrasts are superscripted for both involved trajectories. Contrast tests are not adjusted for mass significance; the chance expected number out of 36 pairwise contrasts at *p* < .05 would be 1.8 per variable, or 3.6 numbered subscripts per table row, in the case of null association. In the presence of a significant overall effect, contrast tests may aid effect direction interpretation; in the absence of a significant overall effect, significance superscripts alone should not be taken as suggesting an effect limited to a few classes.

The contrast tests for predictors with a significant overall effect on TPA generally indicated effects in the expected direction, with risk factors predicting trajectories with higher overall levels, peaks, or endpoints and protective factors predicting in the opposite direction.

### Unadjusted effects of TPA on grade 2 outcomes

Overall effects of TPA on outcomes in isolation tested with a likelihood ratio chi-square with 8 degrees of freedom are shown in the lowermost row of Table 2. TPA significantly predicted externalizing (*R*^*2*^ = 7.4%), academic competence (*R*^*2*^ = 3.6%), and social skills (*R*^*2*^ = 3.7%). Uncorrected for mass significance, pairwise means comparisons among outcomes by TPA with contrast tests are shown in the lower part of Table 3. Regarding externalizing, the no-aggression trajectory (1) had the lowest mean, contrasting with all other trajectories. For academic competence, however, it was the high-stable trajectory (9) that contrasted with all other trajectories.

### Unadjusted effects of early predictors on grade 2 outcomes

When considering the effects of predictors in isolation on any of the four outcomes, 17 predictors—all except maternal and paternal positive parenting and child distress to limitations—influenced one or more of the outcomes. The effects of the significant predictors on each outcome are shown in the rightmost four data columns of Table 2.

### Fully adjusted outcome models with early predictors and TPA

For each distal outcome, we went on to build fully adjusted models for each distal outcome estimating simultaneously all influences, we retained 14 predictors that either had a significant unadjusted effect on TPA (Table 2) or had a significant effect on any of the four outcomes after adjusting for all other predictors in separate multi-predictor models. Thus, paternal education, young father, maternal and paternal sensitivity, and maternal and paternal positive parenting were not included in the fully adjusted models. The findings are shown in Table 4.

**Table 4:** Fully adjusted overall prediction effects of early predictors on trajectory and outcomes, and of trajectory on outcomes.

Predictor	Prediction to trajectory^a^			Prediction to Grade 2 outcomes^b^			
	χ^2^	*p*	Pseudo *R*^a^	Externalizing	Internalizing	Academic competence	Social skills
**Maternal education**	8.2	.417	.08	-.07	-.04	.18***	.10**
**Young mother**	5.2	.732	.07	.04	.04	.08	-.01
**Financial stress**	5.5	.704	.07	.09*	.10*	-.07	-.09*
**Couple relationship (reversed)**	7.8	.453	.08	.03	.08*	-.02	.00
**Single parent**	12.4	.135	.10	.09*	.07	-.09*	-.07
**Maternal depression & anxiety**	10.9	.205	.10	.00	.02	-.02	.00
**Paternal depression & anxiety**	4.2	.834	.06	.03	.00	.00^c^	-.09
**Maternal harsh parenting**	27.8	.001	.16***	.06	.00	-.10	-.05
**Paternal harsh parenting**	20.1	.010	.13**	.02	-.01	-.09	-.05
**Child gender**	49.2	< .001	.21***	.25***	.11**	-.10**	-.27***
**Similar-aged sibling**	90.3	< .001	.28***	-.06	-.06	.02	.06
**Child activity level**	15.2	.055	.12	.05	.06	.03	-.02
**Child distress to limitations**	10.7	.222	.10	-.10*	-.05	.01	.08*
**Child soothability**	11.5	.174	.10	.10*	.04	-.08*	-.10**
**Trajectory** ^d^	—	—	—	.22***	.09	.16	.12

*p < .05

**p < .01

***p < .001.

^a^Comparisons are of a baseline model where all class discrimination effects of all early predictors are included to a reduced model where all the class discrimination effects for a single early predictor are zeroed out completely; prediction to TPA is given as pseudo–*R* as Cohen’s ω derived from robust chi square.

^b^Predictor-to-outcome associations are given as fully standardized regression coefficients.

^c^ We did not allow significant predictor effects to be in the opposite direction of effects in the unadjusted results. This occurred only for the effect of paternal depression and anxiety on academic competence (unadjusted standardized regression coefficient -0.002 [N.S.]; freely estimated fully adjusted standardized regression coefficient 0.139 [p = .018]); the final fully adjusted model for this outcome was thus re-estimated with the effect of this predictor fixed to zero.

^d^Prediction of outcomes from TPA is given as *R*, the square root of *R*^*2*^ computed as the proportional reduction in total outcome variance represented by the residual within class variance, that is, *R*^*2*^ = proportion explained variance = 1 - (residual variance/total variance).

In these adjusted models, the number and magnitude of significant effects, as expected, generally decreased relative to the unadjusted effects (Table 2), highlighting that these adjusted models identify the most proximal predictors of the trajectories and outcomes.

Significant adjusted overall predictors of TPA included the presence of a similar-aged sibling (which did not have a direct effect on any outcome), child gender (which also had direct effects on all outcomes), and maternal and paternal harsh parenting (with no direct effects on outcomes). The upper part of Table 5 displays fully adjusted trajectory prediction contrast coefficients. For the predictors with significant effects, the contrast patterns largely resembled those in the unadjusted results.

**Table 5:** Trajectory predictor coefficient contrasts for fully adjusted effects of early predictors on trajectory and means comparison contrasts for fully adjusted effects of trajectory on outcomes.

Variable	Overall effect^a^	Trajectory								
		1	2	3	4	5	6	7	8	9
Trajectory prediction coefficients with pairwise contrasts for effects of early predictors on trajectory^b^										
**Maternal education**	.08	0.00	0.09	0.10	-0.14	0.18	0.12	0.36	-0.13	-0.24
**Young mother**	.07	0.00	-0.05	0.16	0.18	0.08	-0.17	-0.14	0.18	-0.10
**Financial stress**	.07	0.00	-0.07	0.12	0.11	0.17	0.07	0.25	0.10	0.27
**Couple relationship (reversed)**	.08	0.00^4^	-0.21	0.06^4^	-0.40^1^,^3^,^6^	0.05	0.17^4^	-0.03	-0.13	0.07
**Single parent**	.10	0.00	-0.29^7^	0.24	0.14	0.07	0.15	0.34^2^	0.27	0.15
**Maternal depression & anxiety**	.10	0.00^7^,^9^	0.27	0.34	0.24	0.15	0.19	0.51^1^	0.41	0.71^1^
**Paternal depression & anxiety**	.06	0.00	-0.10	-0.26	0.06	-0.13	-0.14	-0.17	0.03	-0.03
**Maternal harsh parenting**	.16***	0.00^3^,^8^	-0.66^3^,^8^	1.18^1^,^2^,^9^	0.62	0.66	0.72	0.57	1.16^1^,^2^,^9^	0.34^3^,^8^
**Paternal harsh parenting**	.13**	0.00^3^,^6^,^7^,^9^	0.20^7^	0.68^1^	0.51	0.55	0.62^1^	0.76^1^,^2^	0.53	0.80^1^
**Child gender**	.21***	0.00^2^,^3^,^5^,^6^,^7^,^8^,^9^	0.64^1^	0.53^1^	0.39^7^,^9^	0.70^1^	0.80^1^	1.04^1^,^4^	0.69^1^	1.04^1^,^4^
**Similar-aged sibling**	.28***	0.00^3^,^5^,^6^,^7^,^8^,^9^	0.36^7^,^8^,^9^	0.38^1^,^7^,^8^,^9^	0.25^7^,^8^,^9^	0.59^1^	0.49^1^,^7^,^8^,^9^	1.03^1^,^2^,^3^,^4^,^6^	0.97^1^,^2^,^3^,^4^,^6^	1.03^1^,^2^,^3^,^4^,^6^
**Child activity level**	.12	0.00	0.03	-0.26^8^	0.12	0.12	-0.33^8^	0.00	0.20^3^,^6^	0.10
**Child distress to limitations**	.10	0.00^3^,^5^,^6^	0.17	0.48^1^	0.54	0.53^1^	0.45^1^	0.42	0.32	0.46
**Child soothability**	.10	0.00^8^	-0.28	-0.05^8^	0.02^8^	-0.28	-0.21	0.03	-0.49^1^,^3^,^4^	-0.07
Standardized means comparisons with pairwise contrasts for effects of trajectory on distal outcomes^c^										
**Externalizing**	.22***	0.00^2^,^3^,^4^,^5^,^6^,^7^,^8^,^9^	0.33^1^,^7^	0.35^1^,^7^	0.40^1^	0.47^1^	0.62^1^	0.88^1^,^2^,^3^	0.52^1^	0.53^1^
**Internalizing**	.09	0.00	0.17	0.19	0.23	-0.02	0.14	0.16	0.29	0.35
**Academic competence**	.16	0.00^9^	-0.31	-0.20^9^	-0.02^9^	0.07^9^	-0.09^9^	-0.22	-0.27	-0.84^1^,^3^,^4^,^5^,^6^
**Social skills**	.12	0.00	-0.12	-0.13	-0.36	-0.32	-0.31	-0.27	-0.22	-0.55

*p < .05

**p < .01

***p < .001 for overall effects.

^a^Pseudo-*R*/*R* estimate with overall significance test from Table 4.

^b^Trajectory prediction contrast coefficients are fully standardized logistic regression effects on the log odds scale (the effect on the log odds of the class contrast for a change in the predictor of one standard deviation) given relative to membership in Trajectory 1 (the "no aggression" trajectory). Coefficients were standardized by sample standard deviations (Table 1). Contrast coefficients between any pair of trajectories can be derived by subtraction (i.e., for couple relationship [reversed] between Trajectories 6 and 4: 0.17- [-0.40] = 0.57, which is significant at *p* < .05 as indicated by superscripts). A contrast coefficient of 0.69 is equivalent to an odds ratio of 2.0, and a coefficient of -0.69 is equivalent to an odds ratio of 0.5.

^c^The entries are standardized mean differences relative to Trajectory 1.

^123456789^Number superscripts indicate pairwise contrast tests significant at *p* < .05 between the indexed trajectory and the trajectory with the superscripts’ number; significant contrasts are superscripted for both involved trajectories. Contrast tests are not adjusted for mass significance; the chance expected number out of 36 pairwise contrasts at *p* < .05 would be 1.8 per variable, or 3.6 numbered subscripts per table row, in the case of null association. In the presence of a significant overall effect, contrast tests may aid effect direction interpretation; in the absence of a significant overall effect, significance superscripts alone should not be taken as suggesting an effect limited to a few classes

We can thus infer that the set of predictors that were significantly related to TPA (either overall or for some contrasts) prior to adjustment, but not after, may have potentially important *indirect* or *mediated effects*. These predictors include paternal depression and anxiety, maternal education level, financial stress, and young mother status.

In the adjusted models, TPA had a significant overall effect on externalizing (*R*^*2*^ = 5.0%) but not on academic competence (*R*^*2*^ = 2.6%), social skills (*R*^*2*^ = 1.5%) or internalizing (*R*^*2*^ = 0.8%). Adjustment for competing predictors eliminated the significant unadjusted overall effect of trajectory on academic competence and social skills. Of the outcomes with no significant overall effect, for academic competence, large and significant standardized differences indicated a difference involving Trajectory 9 (high-stable), which had the lowest mean, in contrast to Trajectories 1, 3, 4, 5, and 6 (standardized differences ranging from 0.63 to 0.91 standard deviations; individual *p* values for contrasts ranging from .036 to .003). Thus, after adjustment for early predictors, trajectory effects remained significant and important for externalizing (overall) and for academic competence (high-stable in isolation contrasting with five other trajectories) but were eliminated for social skills and remained unrelated to internalizing.

There were direct effects on one or more Grade 2 outcomes of several early predictors including child gender (effects on all 4 outcomes), financial stress (externalizing, internalizing, and social skills), maternal education level (academic competence and social skills), single-parent status (externalizing and academic competence), couple relationship (internalizing), child distress to limitations (three outcomes, all in unexpected direction), and soothability (two outcomes, both in unexpected direction).

## Discussion

Nine latent trajectories represented the development of physical aggression from 1 to 5 years of age. Most of the predictors in the unadjusted analyses were associated with TPA either overall or by significantly contrasting among single trajectories, or significantly associated with at least one Grade 2 outcome, albeit with some notable exceptions. The significant direct effects were fewer and smaller in the fully adjusted model, however those that remained represented the most proximal predictors of TPA and functioning in Grade 2.

### Number and shape of TPA

In contrast to our 9 trajectories, the most comparable trajectory studies have found three to five trajectories [10, 12, 16, 19–22, 24–28]. Our study started earlier than previous research and encompassed the period of peak change (at approximately 2 years of age), had frequent assessment, used a vertically (age) scaled measurement instrument, and utilized 4th-order polynomial growth curves. Indeed, to our knowledge, this study is the only one close to Tremblay’s recommendation [1] regarding assessment frequency. Overall, these qualities provided the power to make finer distinctions and detect more fine-grained developmental patterns of aggression. Nevertheless, there are also important similarities between our results and findings from previous studies. Like other studies [17, 19, 22, 25–27], we identified one high-stable group (Trajectory 9) with the lowest prevalence (3%) and overall poorest outcomes.

The proportion of children belonging to the high-stable trajectory, however, varies across studies. For instance, in accordance with our findings, the SECCYD [22] found a high-stable group from 2 to 9 years of age, constituting 3% of their sample (N = 1195), with the poorest academic and social functioning through 12 years of age [15, 16]. Additionally, Wildeboer et al. [19] identified a high-increasing trajectory across 1.5, 3, and 6 years of age for 3% of the children (N = 4781). Moreover, Joussemet et al. [26] reported a high trajectory group from kindergarten to sixth grade comprising 6% of their sample (N = 1508), whereas Tremblay et al. [12] reported that 13.9% (N = 572) of the participants followed an increasing trajectory from 17 to 42 months of age. Finally, Côté et al. [10] reported that as much as 16.6% (N = 10658) of the participants followed a high-stable trajectory between 2 and 11 years of age, which appeared to include the two highest trajectory groups (i.e., moderate and high) in the SECCYD, explaining the high prevalence.

With respect to the lower end of the aggression spectrum, the SECCYD [22], found a very-low trajectory group accounting for 45% of the children and a low trajectory group accounting for 25%. In our sample, the *low-stable or no aggression group* (1), *medium-peak and low endpoint* groups (2, 3), and *high-start and low-endpoint aggression groups* (4, 5), all with low aggression endpoints at the age of 5 years, albeit with differing developmental patterns, may quantitatively (in all 64%) correspond to some larger very-low or low trajectories, such as those identified in the SECCYD [22]. Such a comparison may be even more reasonable considering that the SECCYD did not start until 2 years of age, after which physical aggression diminishes among most children, particularly those engaging in low levels of aggression.

### Early predictors of TPA (unadjusted)

Our set of nine trajectories revealed more diversity in predictor-trajectory patterns than a less fine-grained classification into fewer developmental patterns might have revealed. All child characteristics (gender and temperament) predicted TPA in the unadjusted results, along with sibling presence, maternal education level, couple relationship, depression and anxiety in both parents, and harsh parenting. Exploration of the finer distinctions in predictor-trajectory effect patterns may provide some clues for what makes children follow particular trajectories. For example, while the intermediate-endpoint and high-stable Trajectories 6–9 shared many predictors, the predictor-trajectory contrasts seemed to suggest some possibly important effect-pattern differences. High maternal harsh parenting and low child soothability were associated with membership in Trajectory 8 (rather than 9, 6, or 7, the other high end point trajectories). This might suggest that Trajectory 8 was somewhat more driven by child temperament and heated mother-child interactions, which is in line with the established knowledge that children’s temperamental characteristics are associated with the development of externalizing problems [60] and with the abovementioned differential susceptibility model postulating that children with difficult temperament are more vulnerable to negative parenting behavior than those with easy temperament [55, 57, 58]. Additionally, higher maternal and paternal education levels were associated with membership in Trajectories 6 and 7 (rather than 8 and 9). This may indicate that parental resourcefulness can function as a protective factor against the high-stable aggression pattern even in the presence of other shared risk factors.

Furthermore, the prediction patterns for the two declining-aggression Trajectories 4 and 5 are interesting to note. Higher child activity level and distress to limitations (relative to Trajectory 1) and high sensitivity in one parent (relative to several other trajectories in the absence of an overall significant predictor effect but with large magnitudes exceeding a log odds change of 0.70 corresponding to an odds ratio of 2.1 for all significant pairwise contrasts, Table 3) predicted membership in these trajectories. This might suggest that parental sensitivity moderates or mitigates aggression development in children with difficult infant temperament, which is in accordance with the differential susceptibility model [55, 57, 58].

### Nonreplicated early predictors of TPA or grade 2 outcomes

Among predictors identified by previous research, neither being a single parent nor being a young mother or father was significantly related to trajectory (although each had direct associations with one or more outcomes in the unadjusted results). The reason for nonreplication is probably the low proportion of single parents in our sample when the child was aged 1 year, as well as the low proportion of young mothers (≤ 25 years old), given the low prevalence of teenage mothers in Norway and the high mean age for primiparous women (i.e., 30.1 years in 2021 [80]).

Parental sensitivity also did not overall predict TPA. This finding merits some attention, not least considering that self-reported harsh parenting practices for mothers and fathers alike were among the strongest predictors of TPA. As previously noted [69], the null findings in the present sample may be partly related to the fact that the parents displayed low levels of aversive or negative parenting behaviors overall during the interactions since the sample was population based (versus clinical), the interactions were short, and the tasks were not designed to induce conflict or elicit negative parenting. Thus, limited variability in the observed parenting behavior may have contributed to the weak associations. This contrasts with the results from the SECCYD [22], which indicated that less sensitive and involved maternal parenting was associated with higher and more stable TPA from toddlerhood to middle childhood. However, that study had access to comprehensive observational data at several ages across infancy and third grade.

### Fully adjusted models

As expected, there were notably fewer significant predictor-trajectory, trajectory-outcome, and predictor-outcome relationships in the fully adjusted set of results than in the unadjusted set. While this may seem dramatic, it is crucial to acknowledge that the fully adjusted model, which is the benchmark test for identifying the most proximal predictors, constituted a rigorous test of all possible simultaneous effects. Importantly, net of all other effects, TPA still predicted externalizing problems and academic competence in Grade 2. Within this context, it may be worth noting that the high-stable trajectory specifically predicted poor academic competence, while the no-aggression trajectory contrasted most strongly with all other groups with respect to low externalizing problems. Taken together, these results both support and extend previous research strongly suggesting that early maternal and paternal harsh parenting practices, child gender, and sibling presence, consistently predict physical aggression development from 1 to 5 years of age after adjustment for all other predictors.

The importance of harsh parenting practices in predicting children’s physical aggression corresponds with previous findings including mothers only [10, 12, 16, 26] and generally with the mentioned theories about the development of aggression and externalizing problems, particularly Patterson’s [45] coercion theory. This is the first study addressing developmental patterns of physical aggression from infancy to preschool age by applying a trajectory approach to link early *paternal* as well as maternal harsh parenting with the development of aggression. Previous research [10, 12, 16, 26] has not explored this association due to a lack of data on fathers’ parenting behaviors. Our results underscore the significance of fathers’ harsh parenting behaviors in infancy for children’s subsequent development of physical aggression and are in line with a limited but growing body of literature highlighting the significance of paternal parenting for children’s and youth’s development [47–48, 81].

In line with the established knowledge that parenting behaviors typically associated with the development of externalizing behaviors tend to coappear with parental psychopathology [82–84], it is theoretically and empirically sound to view the pattern of predictor-trajectory influences in our unadjusted and adjusted results from the perspective that factors such as parental depression and anxiety, couple relationship troubles, and difficult child temperament do not affect aggression development, unless they are associated with an increase in harsh parenting practices.

Regarding *predictor-outcome* effects, early financial stress had a consistent impact on three Grade 2 teacher-rated outcomes (externalizing, internalizing, and social skills). This is notable considering the crude measure used (i.e., a single-item dichotomous self-report). Family financial stress when the child is approximately one year old may thus have long-lasting or continuing effects on social and behavioral functioning. Nevertheless, this may not relate specifically to the development of physical aggression (the predictor had no significant effect on *trajectory* in any set of findings). This result highlights the well-established finding linking socioeconomic conditions to a range of child behavioral outcomes [37, 38]. At the same time, some previous research has shown that low family income is a risk factor for more frequent and stable use of physical aggression across early childhood and adolescence [4, 10, 12]. We have no clear opinion as to why our results did not replicate such findings, but possible explanations could be differences between countries/cultures with a smaller income range and a smaller proportion of families in serious financial hardship in Scandinavia than in North America and Canada, variations in age span, the use of financial stress as an indicator of financial risk as opposed to low family income, and the crude nature of our measure.

### Limitations and suggestions for future research

Our study is limited in several ways, some of which are worth noting. The study design did not permit the disentangling of genetic predispositions and we did not obtain measures of parents’ aggressive or antisocial behavior when they were children or youths. The estimated reliability of the self-reported harsh parenting practices measure was less than desirable. Additionally, due to the study design, only subsets of fathers and mothers provided such reports at 1 year. Under these circumstances, null findings are uninformative, whereas significant results (e.g., prediction of TPA and distal outcomes based on harsh parenting practices) signal true effects that are large enough to be detected with the given sample, although attenuated and less precisely estimated.

The included predictors were all time-invariant; we did not model time-varying or intermediate-timepoint predictor influence as the study progressed (e.g., harsh parenting, financial stress, or new siblings). Finally, as in most previous research, we studied each outcome in isolation, not addressing possible comorbidities or overlaps. However, TPA may well predict certain multivariate outcome configurations or profiles. Similarly, we did not study the effects of profiles of predictors.

Future research should focus on the potentially intricate interplay of predictors, including gender-moderating effects and relevant interaction effects among predictors (e.g., parental depression, harsh parenting practices, child gender and the number of siblings). For example, it is easy to imagine that squabbling among siblings could amplify harsh parenting practices. Research should also address trajectory and predictor effects on profiles of outcomes rather than single outcomes in isolation and effects of predictor profiles rather than of single predictors.

## Conclusions

Understanding common and less common developmental patterns of physical aggression, the link with subsequent social, behavioral, and academic functioning, and markers for the various patterns is crucial for early prevention and intervention efforts. The results of our study confirm and expand previous research in underscoring the importance of addressing harsh parenting practices as an influence on developmental pathways of physical aggression during infancy and toddlerhood. Our findings also support the notion that fathers matter and must be included both in research and early prevention and intervention efforts.

## Supporting information

S1 Appendix Physical aggression questionnaire and telephone interview schedule. (DOCX)

S2 Appendix Scaling of the physical aggression measure. (DOCX)

S3 Appendix Ratings of parent sensitivity from videotaped parent-child structured interaction tasks. (DOCX)

S4 Appendix Attrition. (DOCX)

S5 Appendix Additional comparisons of 2–10 trajectory solutions. (DOCX)

S6 Appendix Details of the 9-trajectory solution. (DOCX)

S1 Fig Loess plot of average child physical aggression by child age. (TIF)

S2 Fig Trajectory plots for 2–9 trajectory solutions. *Note*. Panels A-I graph 4th grade polynomial solutions for the 2 to 10-trajectory solutions, respectively. Age in years centered at 2.2 years is given on the X-axis of each panel. The Y-axis labeled eqtrfe represents the Rasch-scaled physical aggression. The number labels of the trajectories shift as a result of random starts in the estimations, and in the 9-class solution in Panel H the numbering of trajectories is not the same as in the manuscript.
(TIF)
