# Supplementary material for: Modeling trajectories of physical aggression from infancy to pre-school age, their early predictors, and school-age outcomes
Source: PLoS One. 2024 Jun 3;19(6):e0291704. doi: 10.1371/journal.pone.0291704 (PMC11146736; doi:10.1371/journal.pone.0291704)
Supplement: S3 Appendix — (DOCX) [file pone.0291704.s003.docx]

# S4 Appendix: Ratings of parent sensitivity from videotaped parent-child structured interaction tasks

Parent sensitivity was measured based on ratings of videotaped parent-child structured interaction tasks at age 1 year. Fathers were the primarily targeted participants at this age, and more fathers took part in the assessment than mothers, but when fathers were not able or willing to come, the mother took part instead. We thus obtained videotapes of father-child interaction for the greater part of the sample, but for mother-child interaction for a smaller part. The interaction tasks were selected based on their capacity to elicit parent and child behaviors associated with subsequent child adjustment [1, 2] following literature reviews and discussions with international experts. The tasks strived at being gender non-stereotypic and included free-play, where parents were asked to play with their child as they liked with a provided set of toys (4 min.); clean up, where parents were asked to put the toys away and told that the child could help but was not required to (2 min.); structured play, where two sets of toys were presented, a shape sorter box and a set of stacking rings (selected to be too difficult for most 1-year-olds to manage on their own), and parents were asked to help the child as much as they thought necessary with one toy at a time (2 × 3 min.; the assistant informed the dyad when it was time to switch toys). They were also told that there was no need to complete the tasks (e.g., put all stacking rings in the correct order). Prior to the observations, parents were informed that they could choose to discontinue the tasks at any time.

We used the mean of three global ratings - Sensitivity/responsiveness, Detachment/ disengagement (reversed), and Positive regard for the child – from the Norwegian adaptation of the Qualitative Ratings for Parent Child Interaction at 3-15 Months in the NICHD SECCYD study [3, 4] to form a composite index of parent sensitivity. The mean was aggregated across two separately rated interaction segments, one being free-play and clean up, and the other structured play. Each variable was rated on a Likert-type scale of 1 to 5 by trained judges; interrater reliability (single rating, absolute agreement, two-way, random intraclass correlation) ranged from .65 to .74 in the full set of all rating variables [4]. The average was computed if at least three out of six ratings were present, which occurred in 717 fathers and 244 mothers. Internal consistency (alpha) for the composite was .88 for fathers and .86 for mothers in 708 fathers and 242 mothers with complete sets of six ratings.

# References

1. Aspland H, Gardner F. Observational measures of parent-child interaction: An introductory review. Child and Adolescent Mental Health. 2003;8(3),136–143. http://dx.doi.org/10.1111/1475-3588.00061

2. Snyder J, Stoolmiller M, Wilson M, Yamamoto M. Child anger regulation, parental responses to children’s anger displays, and early child antisocial behavior. Social Development. 2003;12*(3)*, 335–360. http://dx.doi.org/10.1111/1467-9507.00237

3. Cox MJ, Crnic K. Qualitative ratings for parent–child interaction at 3–12 months of age. Unpublished manuscript. Department of Psychology, University of North Carolina at Chapel Hill; 2003.

4. Nordahl KB. Early father-child interaction in a father-friendly context: Gender differences, child outcomes, and predictive factors related to fathers’ parenting behaviors with one-year-olds [dissertation]. University of Bergen, Norway; 2014.
