# Supplementary material for: Modeling trajectories of physical aggression from infancy to pre-school age, their early predictors, and school-age outcomes
Source: PLoS One. 2024 Jun 3;19(6):e0291704. doi: 10.1371/journal.pone.0291704 (PMC11146736; doi:10.1371/journal.pone.0291704)
Supplement: S6 Appendix — (DOCX) [file pone.0291704.s006.docx]

**S8 Appendix: Details of the 9-trajectory solution**

Model fit information for the 9-trajectory solution: 54 free parameters; loglikelihood -24757.666, scaling correction factor for MLR 1.4105. Information criteria are given in Table 3 in the manuscript. Class counts and proportions for latent classes based on estimated posterior probabilities are given in Table 1. Final class counts and proportions for classes based on their most likely latent class membership based on 1141 children are given in Table 4 in the manuscript. Table 2 gives the estimated values and standard errors of the trajectory parameters for the 9-trajectory solution. Average latent class probabilities for most likely latent class membership by latent class are given in Table 3. Classification probabilities for the most likely latent class membership by latent class are given in Table 4.

**Table 1. Class counts and proportions for latent classes based on estimated posterior probabilities.**

| **Trajectory** | **Trajectory count** | **Trajectory**  **proportion** |
| --- | --- | --- |
| **1 No aggression** | 1879.36560 | 0.13745 |
| **2 Medium-peak, low-endpoint – flatter** | 2989.32726 | 0.21863 |
| **3 Medium-peak, low-endpoint – early peak** | 2181.64026 | 0.15956 |
| **4 High-start, low-endpoint – lower** | 943.72159 | 0.06902 |
| **5 High-start, low-endpoint – higher** | 734.06290 | 0.05369 |
| **6 Intermediate-endpoint – late flat peak** | 2298.82611 | 0.16813 |
| **7 Intermediate-endpoint – low start** | 1256.48662 | 0.09190 |
| **8 Intermediate-endpoint – high start, high peak** | 1018.82826 | 0.07451 |
| **9 High-stable** | 370.74141 | 0.02711 |

**Table 2. Estimated parameters with associated standard errors of trajectory parameters for the 9-trajectory solution.**

| **Trajectory** | **Trajectory proportion** | **Constant**  **Estimate (SE)** | **Linear**  **Estimate (SE)** | **Quadratic**  **Estimate (SE)** | **Cubic**  **Estimate (SE)** | **Quartic**  **Estimate (SE)** |
| --- | --- | --- | --- | --- | --- | --- |
| **1 No aggression** | .137 | -2.93 (0.13) | 0.15 (0.11) | -0.63 (0.07) | 0.10 (0.07) | 0.02 (0.02) |
| **2 Medium-peak, low-endpoint – flatter** | .219 | -1.75 (0.16) | -0.04 (0.18) | -1.05 (0.08) | 0.58 (0.11) | -0.10 (0.03) |
| **3 Medium-peak, low-endpoint – early peak** | .160 | -0.49 (0.12) | -1.17 (0.15) | -1.26 (0.08) | 1.16 (0.10) | -0.24 (0.03) |
| **4 High-start, low-endpoint – lower** | .069 | -2.17 (0.20) | 0.79 (0.25) | -0.03 (0.14) | -0.82 (0.21) | 0.23 (0.06) |
| **5 High-start, low-endpoint – higher** | .054 | -1.06 (0.17) | 0.90 (0.33) | 0.02 (0.13) | -0.99 (0.22) | 0.27 (0.06) |
| **6 Intermediate-endpoint – late flat peak** | .168 | -0.89 (0.13) | 1.01 (0.15) | -1.15 (0.08) | 0.21 (0.11) | 0.01 (0.03) |
| **7 Intermediate-endpoint – low start** | .092 | 0.49 (0.12) | -0.62 (0.21) | -1.66 (0.10) | 1.45 (0.14) | -0.30 (0.04) |
| **8 Intermediate-endpoint – high start, high peak** | .075 | 0.65 (0.15) | -0.34 (0.23) | -0.84 (0.14) | 0.33 (0.17) | -0.03 (0.04) |
| **9 High-stable** | .027 | 2.01 (0.17) | -0.64 (0.40) | -1.82 (0.18) | 1.49 (0.28) | -0.29 (0.07) |

*Note.* An error variance term of 1.705 (SE 0.029) equal across trajectories and time was estimated.

**Table 3. Average latent class probabilities for the most likely latent class membership (row) by latent class (column).**

| **Trajectory** | **1** | **2** | **3** | **4** | **5** | **6** | **7** | **8** | **9** |
| --- | --- | --- | --- | --- | --- | --- | --- | --- | --- |
| **1 No aggression** | **0.845** | 0.122 | 0.000 | 0.032 | 0.000 | 0.000 | 0.000 | 0.000 | 0.000 |
| **2 Medium-peak, low-endpoint – flatter** | 0.094 | **0.749** | 0.047 | 0.038 | 0.001 | 0.071 | 0.000 | 0.000 | 0.000 |
| **3 Medium-peak, low-endpoint – early peak** | 0.000 | 0.061 | **0.786** | 0.021 | 0.029 | 0.042 | 0.044 | 0.016 | 0.000 |
| **4 High-start, low-endpoint – lower** | 0.034 | 0.125 | 0.041 | **0.724** | 0.061 | 0.015 | 0.000 | 0.000 | 0.000 |
| **5 High-start, low-endpoint – higher** | 0.000 | 0.001 | 0.063 | 0.059 | **0.814** | 0.017 | 0.001 | 0.044 | 0.000 |
| **6 Intermediate-endpoint – late flat peak** | 0.001 | 0.102 | 0.061 | 0.010 | 0.010 | **0.764** | 0.050 | 0.002 | 0.000 |
| **7 Intermediate-endpoint – low start** | 0.000 | 0.000 | 0.060 | 0.000 | 0.004 | 0.057 | **0.807** | 0.055 | 0.017 |
| **8 Intermediate-endpoint – high start, high peak** | 0.000 | 0.000 | 0.017 | 0.000 | 0.031 | 0.001 | 0.049 | **0.879** | 0.023 |
| **9 High-stable** | 0.000 | 0.000 | 0.000 | 0.000 | 0.000 | 0.000 | 0.020 | 0.056 | **0.924** |

**Table 4. Classification probabilities for the most likely latent class membership (column) by latent class (row).**

| **Trajectory** | **1** | **2** | **3** | **4** | **5** | **6** | **7** | **8** | **9** |
| --- | --- | --- | --- | --- | --- | --- | --- | --- | --- |
| **1 No aggression** | **0.832** | 0.151 | 0.000 | 0.016 | 0.000 | 0.001 | 0.000 | 0.000 | 0.000 |
| **2 Medium-peak, low-endpoint – flatter** | 0.076 | **0.756** | 0.045 | 0.038 | 0.000 | 0.085 | 0.000 | 0.000 | 0.000 |
| **3 Medium-peak, low-endpoint – early peak** | 0.000 | 0.065 | **0.788** | 0.017 | 0.020 | 0.069 | 0.033 | 0.008 | 0.000 |
| **4 High-start, low-endpoint – lower** | 0.063 | 0.122 | 0.050 | **0.695** | 0.042 | 0.027 | 0.000 | 0.000 | 0.000 |
| **5 High-start, low-endpoint – higher** | 0.000 | 0.003 | 0.088 | 0.075 | **0.751** | 0.035 | 0.007 | 0.042 | 0.000 |
| **6 Intermediate-endpoint – late flat peak** | 0.000 | 0.093 | 0.040 | 0.006 | 0.005 | **0.826** | 0.030 | 0.000 | 0.000 |
| **7 Intermediate-endpoint – low start** | 0.000 | 0.001 | 0.077 | 0.000 | 0.001 | 0.099 | **0.779** | 0.038 | 0.006 |
| **8 Intermediate-endpoint – high start, high peak** | 0.000 | 0.000 | 0.035 | 0.000 | 0.029 | 0.005 | 0.066 | **0.845** | 0.020 |
| **9 High-stable** | 0.000 | 0.000 | 0.000 | 0.000 | 0.000 | 0.000 | 0.055 | 0.060 | **0.885** |

Table 5 shows model-estimated and pseudo-class assigned (i.e., by children’s highest estimated trajectory membership probability) proportions of trajectories, and the distribution of boys and girls within trajectories. The estimated and assigned proportions were overall very similar, supporting the model’s fit to the empirical distinctions present in the data set. Gender differences in development were clear in that more boys than girls were described by the intermediate-endpoint and high-stable Trajectories 6-9 (from 60% to a maximum of 69% pseudo-class assignments for Trajectory 9), and more girls than boys were described by the no-aggression Trajectory 1 (28% boys by pseudo-class assignments). It is noteworthy that there were no trajectories that exclusively described only one gender; the low-endpoint Trajectories 2-5 described more or less even proportions of boys and girls, and each trajectory, including those with the most uneven gender distribution, described a fair proportion of the less represented gender (1 boy per 2.5 girls in Trajectory 1, and 1 girl per 2.2 boys in Trajectory 9).

**Table 5.** **Trajectory proportions and gender proportions within trajectory.**

|  | **Model estimates** | |  | **Pseudo-class assignments** | | | |
| --- | --- | --- | --- | --- | --- | --- | --- |
| **Trajectory** | **Trajectory proportion** | **Proportion  boys within trajectory^a^** |  | **Girls** | **Boys** | **Trajectory proportion** | **Proportion boys within trajectory** |
| **1 No aggression** | .137 | .24 |  | 111 | 44 | .136 | .28 |
| **2 Medium-peak, low-endpoint – flatter** | .219 | .51 |  | 130 | 126 | .224 | .49 |
| **3 Medium-peak, low-endpoint – early peak** | .160 | .51 |  | 89 | 98 | .164 | .52 |
| **4 High-start, low-endpoint – lower** | .069 | .45 |  | 39 | 34 | .064 | .47 |
| **5 High-start, low-endpoint – higher** | .054 | .58 |  | 24 | 32 | .049 | .57 |
| **6 Intermediate-endpoint – late flat peak** | .168 | .62 |  | 80 | 119 | .174 | .60 |
| **7 Intermediate-endpoint – low start** | .092 | .66 |  | 35 | 64 | .087 | .65 |
| **8 Intermediate-endpoint – high start, high peak** | .075 | .59 |  | 35 | 52 | .076 | .60 |
| **9 High-stable** | .027 | .70 |  | 9 | 20 | .025 | .69 |

^a^The model estimated proportion of boys was derived from the set of unstandardized and unadjusted trajectory prediction contrast coefficients for child gender as trajectory-membership predictor (Table 3 in the main article displays the corresponding standardized set) and the model estimated trajectory proportions (pseudo-class proportions yielded the same result).
