# Supplementary figures and images for: Modeling trajectories of physical aggression from infancy to pre-school age, their early predictors, and school-age outcomes
Source: PLoS One. 2024 Jun 3;19(6):e0291704. doi: 10.1371/journal.pone.0291704 (PMC11146736; doi:10.1371/journal.pone.0291704)

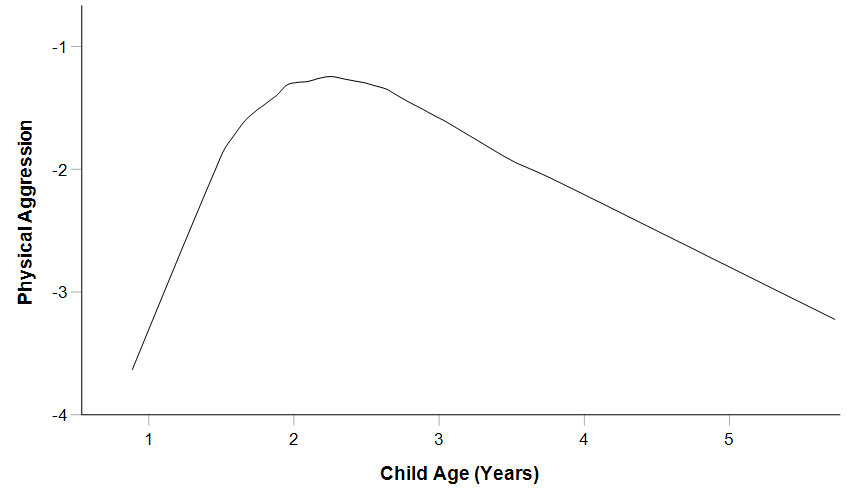

Supplement: S1 Fig — (TIF) [file pone.0291704.s007.tif]

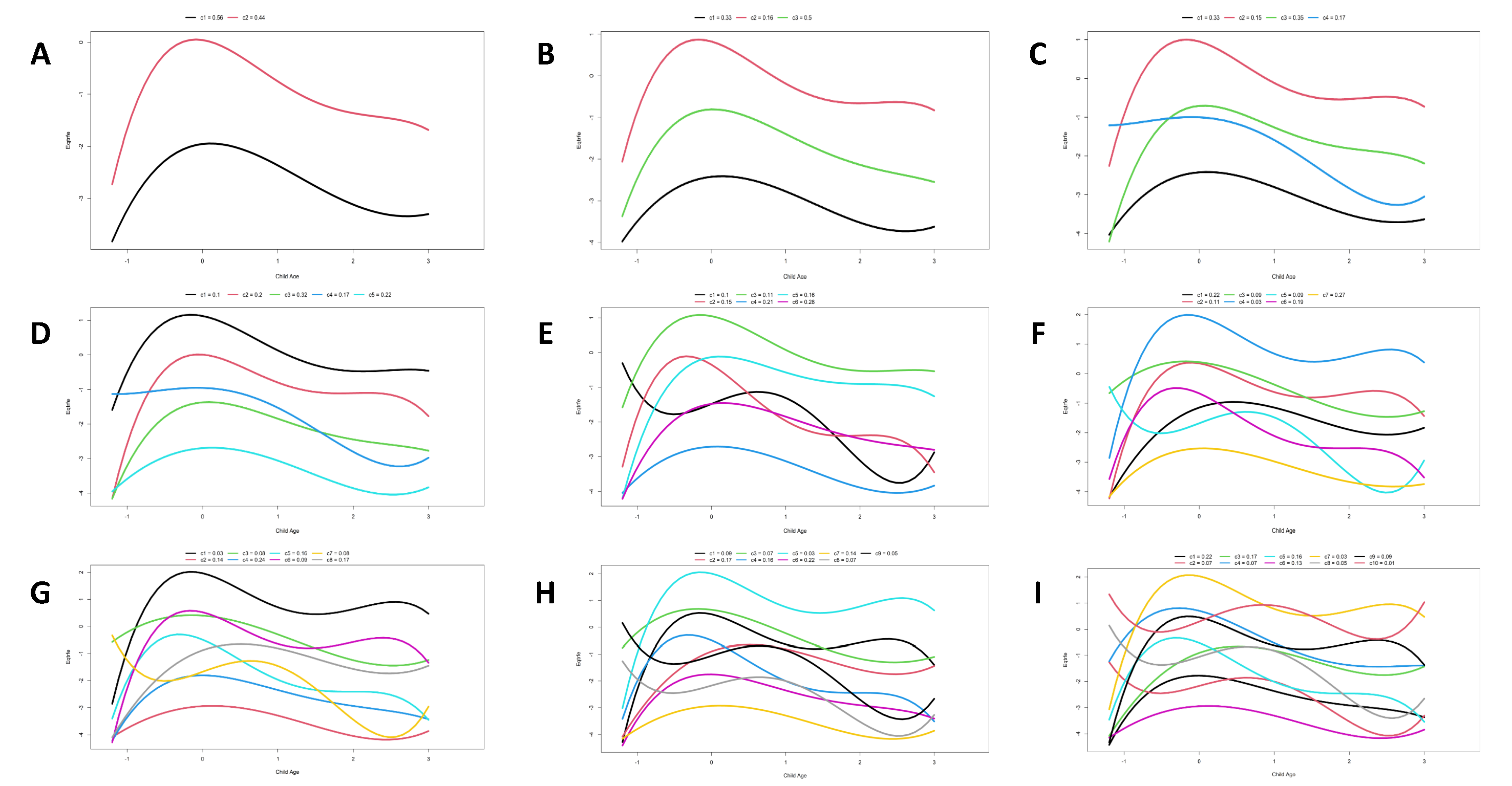

Supplement: S2 Fig — Note. Panels A-I graph 4th grade polynomial solutions for the 2 to 10-trajectory solutions, respectively. Age in years centered at 2.2 years is given on the X-axis of each panel. The Y-axis labeled eqtrfe represents the Rasch-scaled physical aggression. The number labels of the trajectories shift as a result of random starts in the estimations, and in the 9-class solution in Panel H the numbering of trajectories is not the same as in the manuscript. (TIF) [file pone.0291704.s008.tif]
